# SCEMENT: scalable and memory efficient integration of large-scale single-cell RNA-sequencing data

**DOI:** 10.1093/bioinformatics/btaf057

**Published:** 2025-02-22

**Authors:** Sriram P Chockalingam, Maneesha Aluru, Srinivas Aluru

**Affiliations:** Institute for Data Engineering and Science, Georgia Institute of Technology, Atlanta, GA-30332, United States; School of Biological Sciences, Georgia Institute of Technology, Atlanta, GA-30332, United States; School of Computational Science and Engineering, Georgia Institute of Technology, Atlanta, GA-30332, United States

## Abstract

**Motivation:**

Integrative analysis of large-scale single-cell data collected from diverse cell populations promises an improved understanding of complex biological systems. While several algorithms have been developed for single-cell RNA-sequencing data integration, many lack the scalability to handle large numbers of datasets and/or millions of cells due to their memory and run time requirements. The few tools that can handle large data do so by reducing the computational burden through strategies such as subsampling of the data or selecting a reference dataset to improve computational efficiency and scalability. Such shortcuts, however, hamper the accuracy of downstream analyses, especially those requiring quantitative gene expression information.

**Results:**

We present SCEMENT, a SCalablE and Memory-Efficient iNTegration method, to overcome these limitations. Our new parallel algorithm builds upon and extends the linear regression model previously applied in ComBat to an unsupervised sparse matrix setting to enable accurate integration of diverse and large collections of single-cell RNA-sequencing data. Using tens to hundreds of real single-cell RNA-seq datasets, we show that SCEMENT outperforms ComBat as well as FastIntegration and Scanorama in runtime (upto 214× faster) and memory usage (upto 17.5× less). It not only performs batch correction and integration of millions of cells in under 25 min, but also facilitates the discovery of new rare cell types and more robust reconstruction of gene regulatory networks with full quantitative gene expression information.

**Availability and implementation:**

Source code freely available for download at https://github.com/AluruLab/scement, implemented in C++ and supported on Linux.

## 1 Introduction

Several different methodologies have been developed for integrating multiple single-cell RNA-sequencing (scRNA-seq) datasets, with the aim of eliminating batch effects inherent in samples spanning different locations, labs, and conditions, while also conserving biological variation. Currently available scRNA-seq integration methods can be classified into three major categories: (i) methods that output embedding onto a reduced dimensional space such as PCA (Korsunsky *et al.* 2019, Xu *et al.* 2021), (ii) methods that output graphs such as a cell–cell k-nearest-neighbor graph (Haghverdi *et al.* 2018, Polański *et al.* 2020), and (iii) methods that retain the gene-level quantitative information, i.e. their output is a gene expression matrix containing gene expression profiles from input cells (Johnson *et al.* 2007, Zhang *et al.* 2019). Although these methods have been useful in integrating single-cell datasets generated under a variety of tissues and conditions, their applicability is constrained by limitations on scale of data they could handle, and thus integration of large numbers of cells and complex scRNA-seq datasets still remains a challenge.

A recent comprehensive survey of 16 different supervised and unsupervised scRNA-seq integration methods showed that none of these methods could successfully integrate 970 K cells collected from multiple samples of a mouse brain dataset due to runtime and memory constraints (Luecken *et al.* 2022). To improve computational efficiency and scalability, a few tools designed for large data integration perform one or more of the following: (i) partition the data at discrete steps of the processing pipeline to solve data-specific problems (Li *et al.* 2022a), (ii) operate on a reduced dimensional space of cells (Haghverdi *et al.* 2018, Korsunsky *et al.* 2019), (iii) use only a representative subset of datasets/genes (such as a reference dataset or a few highly variable genes) (Dhapola *et al.* 2022, Hao *et al.* 2024), and (iv) use unscaled data instead of scaled data to avoid generating a dense matrix (Luecken *et al.* 2022). However, such approaches limit the applicability of the integrated data for downstream processing steps, such as for reconstruction of robust gene regulatory and cell–cell interaction networks, as inclusion of only a subset of genes and/or datasets for gene–gene and cell–cell inference leads to an approximate network that may not be suitable for studying subtle and rare interactions (Bansal *et al.* 2007, Belcastro *et al.* 2011, Bafna *et al.* 2023). Our goal is to overcome limitations on numbers of genes or cells, while simultaneously achieving data size scalability and adequate performance. Here, we present a novel approach that uses a sparse implementation of an empirical Bayes-based linear regression model to integrate scRNA-seq data from a large number of datasets and expression profiles. While the concept of applying a linear regression model is well established in various biological research areas (Kerr 2003, Pierson and Yau 2015, Dirmeier *et al.* 2018), including in single-cell research (Johnson *et al.* 2007), our method SCEMENT (SCalablE and Memory-Efficient iNTegration) incorporates multiple algorithmic improvements for a faster and efficient way to enable large-scale scRNA-seq data integration with millions of cells and tens of thousands of genes.

In this paper, we demonstrate that by engineering sparsity during all computations, even in cases where mathematical expressions involving the input sparse matrix X could potentially lead to intermediate dense matrices, and by designing an efficient order of computations, SCEMENT outperforms ComBat (Johnson *et al.* 2007), FastIntegration (Li *et al.* 2022a), and Scanorama (Hie *et al.* 2019) in run-time (upto 214× faster) and memory usage (upto 17.5× less). It performs batch correction and integration of 4 million cells collected from 121 samples with more than 38 K genes in just 22 min. In addition, SCEMENT not only maintains meaningful biological gene expression variations across cell types even when cells are clustered by their condition, but also facilitates downstream processing of single-cell data for better identification of rare cell types and more robust reconstruction of gene networks with full gene expression information.

The paper is organized as follows: Section 2 describes key steps used in the SCEMENT method for large-scale integration of single-cell data, and optimizations achieved in each step to make it faster and more memory-efficient compared to other methods. Section 3 describes experimental results to demonstrate improvements in quality and scalability, and also SCEMENT’s utility for downstream applications: discovery of rare cell types and more robust gene network reconstruction.

## 2 Methods

Linear regression models provide two key advantages for the integration of gene expression profiles from scRNA-seq data: (i) the ability to accommodate various experimental conditions and parameters and (ii) the ability to retain quantitative gene expression values after integration. These methods are also more amenable to parallelization and optimization when compared to the graph-based algorithms used in other scRNA-seq integration methods, such as Seurat and Scanorama (Wang *et al.* 2021). To account for technical and biological variations resulting from different conditions, a linear model uses numerical and categorical type variables to represent these variations. In this work, we assume that a given dataset includes only categorical variables, as is common with single-cell RNA-seq datasets.

### 2.1 SCEMENT’s approach for large-scale integration

**Figure 1.**
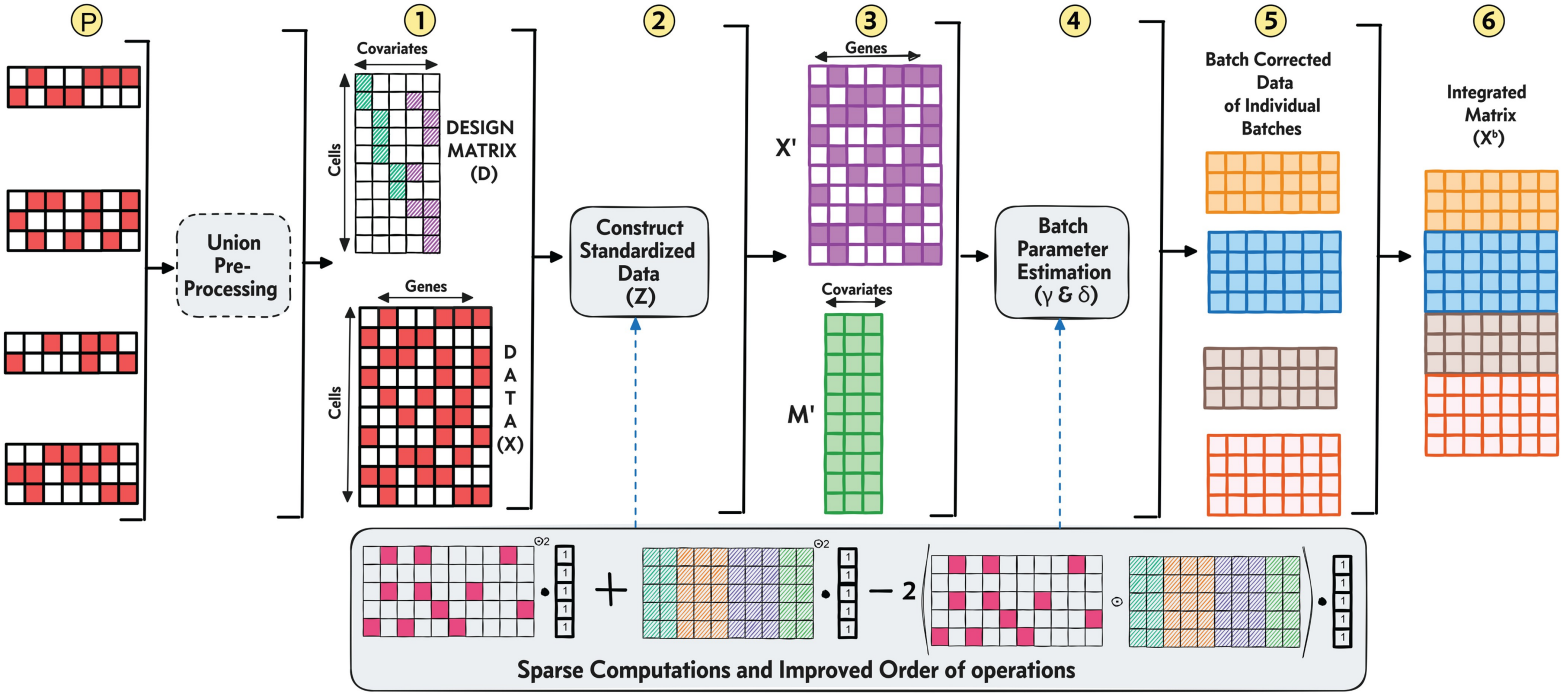
Overall workflow of SCEMENT. A detailed description of the integration methodology is provided in Section 2.1.

Similar to the model outlined in ComBat (Johnson *et al.* 2007), we start with the following generative empirical Bayes-based linear regression model:

X=α+Dβ+γ+δϵ

where the gene expression data X of m cells and n genes is modeled as a linear function of four terms:

- The average or overall gene expression, denoted by α, is an m×n matrix. Each row i in α corresponds to the gene expression profile of the set of conditions the cell i belongs to.
- Linear combination of the independent variables, β. It is the matrix of regression coefficients (size c×n) with each column corresponding to a specific variable. In case of integration, the independent variables are the condition/batch variables. D is the design matrix, a binary m×c matrix such that entry *D[i, j]* is 1 if input i is observed under the condition j (Step 1 of Fig. 1).
- Additive batch effect denoted by γ (a matrix of size m×n).
- Multiplicative batch effect denoted by δ (a matrix of size m×n). Furthermore, ϵ, a matrix of size n×n, is the error term, assumed to follow a normal distribution with variance σ, i.e., $\epsilon ∼N(0,\sigma^{2}I_{n})$.

SCEMENT employs an efficient algorithm to estimate the model parameters: α, β, γ, δ, and σ. Complete details of the empirical Bayes solution are provided in the supplementary text, available as supplementary data at *Bioinformatics* online. In this section, we present the key contributions underlying SCEMENT that enable large-scale integration. As is common in statistics, we use $\hat{\alpha }$, $\hat{\beta }$, $\hat{\gamma }$, $\hat{\delta }$, and $\hat{\sigma }$ to represent the estimated values of α, β, γ, δ, and σ. The overall approach incorporated in the SCEMENT algorithm is illustrated in Fig. 1.

#### 2.1.1 SCEMENT’s strategy for computing the standardized matrix

Integration of gene expression data starts with the computation of the standardized matrix Z (see Supplementary text S1.1.1, available as supplementary data at *Bioinformatics* online, for how Z is defined and computed) from the input data matrix X, since Z provides well-behaved mean and variance characteristics for more robust analyses (Step 2 of Fig. 1). Computing Z requires computation of $\hat{\alpha }$, $\hat{\beta }$, and the variance $\hat{\sigma }$, and these require matrix computation operations involving the data matrix and the design matrix. For microarray and bulk RNA-seq datasets, where the number of observations are in the order of thousands, currently available dense matrix multiplication routines are sufficient. However, for single-cell RNA-seq datasets with hundreds of thousands to millions of cells, computation of Z necessitates a memory-efficient approach.

**Computing**  $\hat{\alpha }$  **and**  $\hat{\beta }$**:** We propose a space- and time-efficient method to accelerate the computation of $\hat{\alpha }$, with the following optimization strategies that enable scaling to millions of cells and tens of thousands of genes.

1. Current implementations use a 32-bit integer matrix to represent the design matrix D. When all the β variables are only categorical, entries of D are binary, i.e. either 1 or 0 and hence do not require 32-bit representation. We use 8 bits for entries in D to improve space by a factor of 4, which also leads to time efficient computation of $D^{T}D$.
2. For computing $\hat{\beta }={\left(D^{T}D\right)}^{-1}D^{T}X$, the order of multiplying the matrices can have significant effect on the runtime. For real datasets, since the number of observations is an order of magnitude larger than the number of genes, multiplication of ${\left(D^{T}D\right)}^{-1}$ with $D^{T}X^{T}$ is a better way to compute $\hat{\beta }$ when compared to the product of ${\left(D^{T}D\right)}^{-1}D^{T}$ and $X^{T}$ because the former takes $O(c^{2})$ time, while the latter takes $O(c^{2}m)$ time when m≫n.

Subsequent to computing $\hat{\beta }$, $\hat{\alpha }=\frac{1}{n}1_{m}\cdot (N_{c}^{T}\hat{\beta })$ is computed by using a simple matrix vector multiplication routine.

**Computing**  $\hat{\sigma }$**:** Computing ${\hat{\sigma }}^{2}=\frac{1}{m}{\left(X-D^{T}\beta \right)}^{⊙2}\cdot 1_{m}$ can lead to explosion in the amount of memory usage even when X is sparse, because realizing $X-D^{T}\beta$ in-memory creates a dense intermediate matrix of size m×n.

Let $R=D^{T}\beta$. To avoid realizing this intermediate matrix X−R, SCEMENT employs the algebraic expansion ${\left(X-R\right)}^{⊙2}\cdot 1_{m}=X^{⊙2}\cdot 1_{m}+R^{⊙2}\cdot 1_{m}-(2X⊙R)\cdot 1_{m}$ (similar to ${\left(a-b\right)}^{2}=a^{2}+b^{2}-2ab$). By expanding ${\left(X-R\right)}^{⊙2}$ into three terms, SCEMENT computes the three terms efficiently as follows.

1. The first term $X^{⊙2}\cdot 1_{m}$ is computed by squaring each entry in sparse X summing it row-wise. Since X is a sparse matrix, this can be computed using sparse matrix routines.
2. To compute the second term, we exploit the unique property of the matrix R that R has as many unique rows as the number of unique condition profiles. A condition profile is the set of unique conditions a cell can belong to. Even in a large collection of datasets comprising millions of cells, the number of unique condition profiles is limited to few dozens, guaranteeing sparsity.
  For efficient computation, we exploit the fact that all the cells that belong to the same condition profile have the same row vector in D and, therefore in R. In order to compute $R^{2}\cdot 1_{m}$ efficiently, SCEMENT first enumerates all the unique condition profiles D and then computes the product vector ($D_{\left(i,\cdot \right)}\cdot \hat{\beta }$) separately for each one of them. We accomplish this with the aid of two auxiliary data structures: (i) a configuration matrix G, which contains set of condition-profile vectors and (b) a condition-profile lookup vector $l_{D}$. $R^{⊙2}\cdot 1_{m}$ can be computed by adding these vectors as many times as the number of cells in the corresponding condition profiles.
3. The condition-profile lookup vector $l_{D}$ and the configuration matrix G can also be used to compute the third term efficiently. SCEMENT employs a series of multiplication of sparse matrix and dense vector, one for each unique condition profile.

With our efficient way of computing these three terms, the partial sums corresponding to individual condition profiles are added as soon as they are computed, thus saving memory and time.

Finally, in order to maintain low memory footprint, we do not fully realize Z as $\left(X-{\hat{\alpha }}^{T})/({\hat{\sigma }}^{2}\cdot 1_{n}\right)$. We retain the left-hand side ($X/({\hat{\sigma }}^{2}\cdot 1_{n})$) and the right-hand side (${\hat{\alpha }}^{T}/({\hat{\sigma }}^{2}\cdot 1_{n})$) of Z as matrices $X^{\prime }$ and $M^{\prime }$, respectively (Step 3 of Fig. 1).

Each of the above steps to compute ${\left(X-R\right)}^{⊙2}$ can be accomplished efficiently in parallel as follows. For the first term $X^{⊙2}\cdot 1_{m}$, each of the row sum of squares can be computed by a parallel reduction across each row $X^{⊙2}$. In case of the second term, a coordinate sparse (COO) representation of the matrix X, along with the row-wise distribution of configuration matrix G and the look-up vector $l_{D}$, enables efficient distribution of the row-wise computations in parallel. Similarly, parallel computation of the third term is accomplished by row-wise distribution of the configuration matrix G.

Note that $X^{\prime }$ is the same size as the input matrix and $M^{\prime }$ is of the size $c^{\prime }\times n$, where $c^{\prime }$ is the number of unique configuration profiles in the dataset. In other words, $M^{\prime }$ has only one row only for each unique configuration profile and not for each cell. In the next sections, we show how $X^{\prime }$ and $M^{\prime }$ matrices can be used in the downstream computations instead of the Z matrix.

#### 2.1.2 SCEMENT’s approach for batch correction

**Iterative**  $\hat{\gamma_{i}}$  **and**  ${\hat{\delta }}_{i}$  **update:** Empirical Bayes method follows an iterative algorithm to estimate $\hat{\gamma_{i}}$ and ${\hat{\delta }}_{i}$. The primary challenge in the iterative update of ${\hat{\delta }}_{i}^{\left(k+1\right)}$ at the k-th iteration is to evaluate the expression

$${\left(Z-{\hat{\gamma }}_{i}^{{\left(k+1\right)}^{T}}\cdot 1_{n}\right)}^{⊙2}\cdot 1_{n}$$

without realizing the dense matrix Z in memory, where ${\hat{\gamma }}_{i}^{\left(k+1\right)}$ is the update of $\gamma_{i}$ at k-th interation (Step 4 of Fig. 1).

Similar to the computation of ${\hat{\sigma }}^{2}$ discussed in Section 2.1.1, the above computation can be accomplished by expanding the expression into three terms (i) $Z^{⊙2}\cdot 1$, (ii) ${\hat{\gamma }}_{i}^{{\left(k+1\right)}^{T}}\cdot 1$, and (iii) $Z⊙{\hat{\gamma }}_{i}^{{\left(k+1\right)}^{T}}\cdot 1$. As mentioned earlier, in SCEMENT, Z is maintained as two matrices $X^{\prime }$ and $M^{\prime }$. Therefore, the first and the third terms expand to $(X^{\prime }-M^{\prime })⊙2\cdot 1$ and $(X^{\prime }-M^{\prime })⊙{\hat{\gamma }}_{i}^{{\left(k+1\right)}^{T}}\cdot 1$. Both these terms can be further expanded, and each of the individual terms can be computed without having to realize the standardized Z matrix. Each term is successively added up to obtain the update for ${\hat{\delta }}_{i}^{\left(k+1\right)}$.

By not directly computing the Z matrix and using algebraic expansion for the terms where Z appears, we retain sparsity of the computations, and thereby efficiently compute each update. Also, parallel computation of these terms is accomplished in a similar manner to the computations described in Section 2.1.1.

**Batch-corrected matrix:** In the final step, we convert the sparse X matrix to dense and update it as the batch-corrected matrix $X^{b}=\hat{\alpha }+X\hat{\beta }+\frac{\hat{\sigma }}{\hat{\delta }}(Z_{i}-\hat{\gamma_{i}})$. Similar to computation of Z in Section 2.1.1, it is possible to retain the batch-corrected matrix, $X^{b}$, as two sparse matrices—one each corresponding to the left-hand side and the right-hand side of Z (Step 5 of Fig. 1). However, to facilitate downstream processing of the integrated matrix, such as for computing PCA, UMAP, t-SNE, clustering, and plotting, SCEMENT converts the sparse X to dense X (Step 6 of Fig. 1).

### 2.2 SCEMENT’s implementation

We implemented two versions of SCEMENT compatible with the *AnnData* data structure used in *Scanpy*. One in the *python* programming language (pySCEMENT) and the second is a faster parallel version in *C++* (SCEMENT-CPP). Both versions use single precision floating point (32-bit) values for the computations. The *python* version uses sparse matrix libraries available in the *scipy* python package for representing the input data and $X^{\prime }$. Though the input data and $X^{\prime }$ are stored in compressed sparse array representation, *numpy* library arrays are used to store all the other matrices and vectors in the algorithm. For the construction of design matrix D, we used the *formulaic* library, which allows for saving space with the use of 8-bit integers.

OpenMP is used for implementation of the parallel SCEMENT algorithm in *C++*. In order to enable efficient computations, we use the coordinate sparse (COO) representation to store the input data and $X^{\prime }$. *Armadillo* C++ libraries are used for representing all other dense vectors and matrices. While parallel sparse computations are implemented as per Section 2.1, *ScaLAPACK* library is used for computations involving dense matrices and vectors.

It should be noted that in contrast to existing integration methods, SCEMENT provides an optional pre-processing step (shown as Step P of Fig. 1). This step allows for construction of an integrated data matrix containing the union of genes across all batches. For each sample/batch, we first identify genes missing in that particular sample/batch, but present in any of the other batches. We then insert rows with zero entries corresponding to the missing genes into each of the gene expression matrices such that all of the input matrices have the same set of genes. Subsequent merging of the modified matrices thus generates an integrated data matrix containing the union of genes.

### 2.3 Performance assessment of SCEMENT

We performed two types of evaluation studies using real scRNA-seq datasets from different tissues and organisms (Table S1, available as supplementary data at *Bioinformatics* online). First, we compared performance of four other previously published integration methods—FastMNN, ComBat, Scanorama, and Seurat (Table S1, available as supplementary data at *Bioinformatics* online) in terms of both integration quality and separation of clusters in UMAP plots using scRNA-seq datasets from *Arabidopsis thaliana* plant root (Jean-Baptiste *et al.* 2019, Gala *et al.* 2021) and human aortic valve (Xu *et al.* 2020). Results from these runs were subsequently used to compute quality control metrics according to the “scIB” software package (Luecken *et al.* 2022). We also visually compared clusters of aortic valve dataset generated using SCEMENT, with the aforementioned four methods. Cell-type annotations used in UMAP plots of human aortic valve cells were according to Xu *et al.* (2020), and for Arabidopsis cells according to Jean-Baptiste *et al.* (2019) and Gala *et al.* (2021).

Next, we evaluated scalability of the integration methods using two different human peripheral blood mononuclear cell (PBMC) datasets: (i) a COVID-19 dataset of ∼1.23 million cells from 205 samples (Ren *et al.* 2021) and (ii) a collection of 17 human PBMC datasets containing a total of 794,170 cells obtained from the 10× Genomics web repository (Table S2, available as supplementary data at *Bioinformatics* online). We assessed runtime and memory usage of all the integration methods with varying number of datasets/cells, and with integrated data consisting of union as well as intersection of genes. All runs were conducted on a machine equipped with a 72-core Intel^®^ Xeon^®^ E7-8870 CPU and main memory of 1 TB shared between all the cores.

#### 2.3.1 Construction and analysis of gene regulatory networks

We used the pySCENIC workflow (Kumar *et al.* 2021) to construct gene regulatory networks (GRNs) from integrated data matrices consisting of union as well as intersection of genes. Here, we use human PBMC datasets (Table S2, available as supplementary data at *Bioinformatics* online) containing cells ranging from ≈ 20 000 to 166 000 cells. The quality and performance of the resulting networks were assessed using standard statistical measures: recall (percentage of correct edges predicted), precision (percentage of correct edges among all edges inferred), the F-score defined as: F-score = (2×precision×recall)/(precision+recall), and the area under the receiver operating characteristic (AUROC) and the area under the precision-recall (AUPR) curves plotted by comparing reconstructed network(s) against the reference network. To evaluate the biological relevance of networks generated from different integrated matrices, we used known human transcriptional regulatory reference networks from the TRRUSTv2 database (Han *et al.* 2018), hTFtarget database (Zhang *et al.* 2020), and PBMC (Li *et al.* 2022b) as ground truths. These networks were constructed by text mining of published literature and manual curation and include a total of 1642 non-redundant high confidence regulatory interactions between 168 transcription factors (TFs) and 842 target genes (Table S3, available as supplementary data at *Bioinformatics* online). For the purpose of computing statistical measures, all known TF–target interactions from amongst the 1642 interactions were considered as true positives, whereas TF–target interactions not listed in the ground truth network were considered as true negatives.

#### 2.3.2 Cell-type identification from large-scale integrated data

We generated integrated data matrices with SCEMENT-CPP using varying number of datasets and cells sampled from the ≈1.2 million cell scRNA-seq dataset (Ren *et al.* 2021) and subsequently applied the Azimuth package (https://azimuth.hubmapconsortium.org/) on the integrated data to automatically identify various human PBMC cell-type populations.

## 3 Results and discussion

### 3.1 Linear regression model for scRNA-seq integration

SCEMENT is designed to be an unsupervised computational method that makes large-scale batch correction and integration of scRNA-seq datasets feasible, while retaining gene expression profiles of all available genes from input cells in the integrated data matrix. A recent survey of over 16 different supervised and unsupervised scRNA-seq integration methods (Luecken *et al.* 2022) ranked four unsupervised methods—FastMNN (Haghverdi *et al.* 2018), Seurat v3 (Butler *et al.* 2018), Scanorama (Hie *et al.* 2019), and ComBat (Johnson *et al.* 2007), amongst the top 10 best performing methods. These four methods also meet our criteria of returning an integrated gene expression matrix of batch corrected values as output. Therefore, we sought to further evaluate FastMNN, Scanorama, Suerat, and ComBat.

We used scRNA-seq datasets generated from two different organisms containing varying sizes and complexity to assess the four integration methods: wild-type *A. thaliana* plant root dataset (AtRD) with 14 427 cells collected from two separate studies and nine different batches (Jean-Baptiste *et al.* 2019, Gala *et al.* 2021), and a human aortic valve dataset (HAVD) containing 17 985 cells collected from four individuals; two healthy and two diseased (Xu *et al.* 2020).

**Figure 2.**
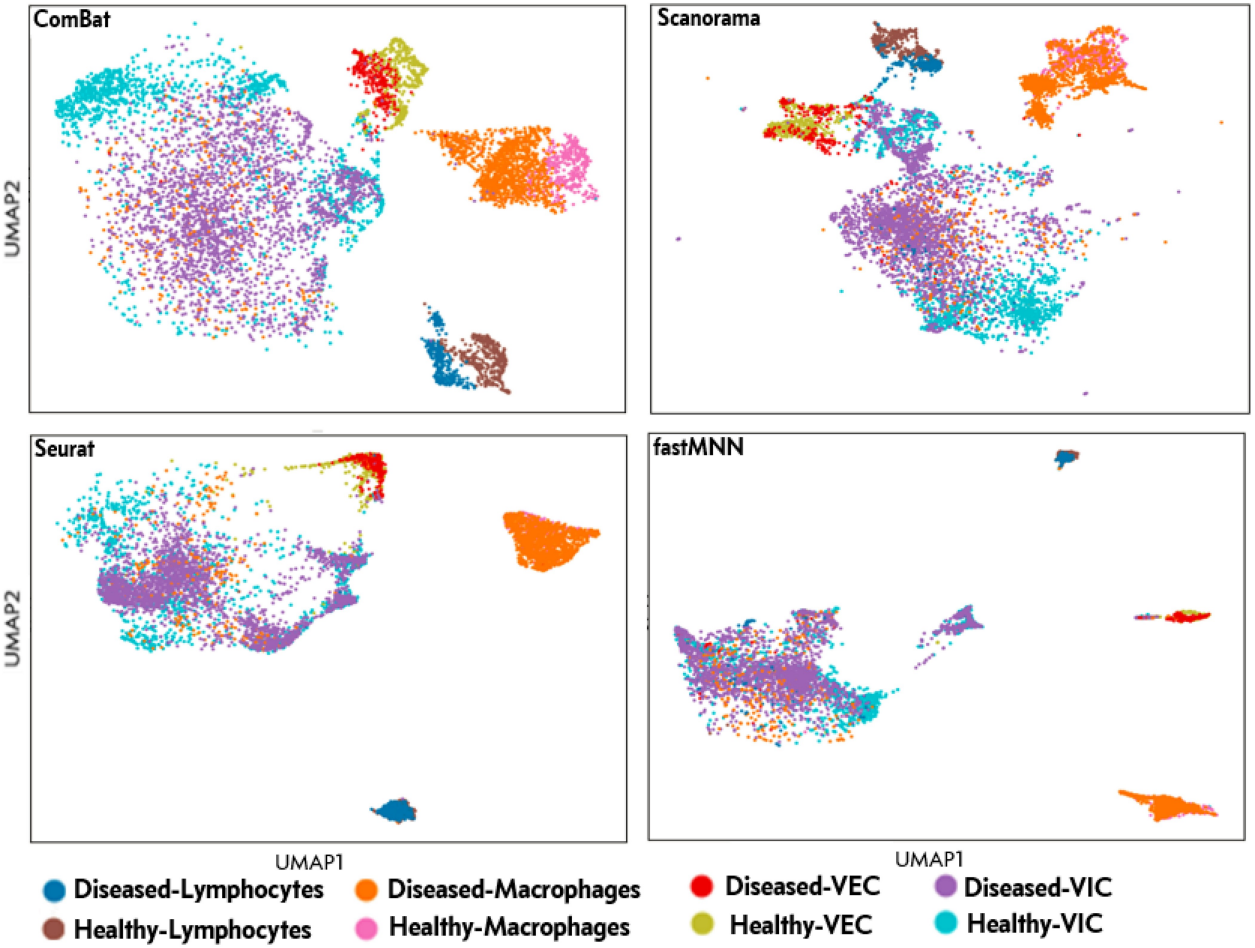
UMAP visualizations of cell-type clusters from the human aortic valve dataset after batch correction and integration with ComBat, Scanorama, Seurat, and FastMNN.

We employed eight different evaluation metrics from the scIB package (Luecken *et al.* 2022) in conjunction with UMAP visualizations to make valid comparisons of the four integration methods. Our results show that ComBat’s overall performance is slightly superior compared to the other three methods for the AtRD datasets and is similar to Scanorama and FastMNN but inferior to Seurat with respect to the HAVD dataset (Table 1). The UMAP visualizations, however, suggest that the ComBat model is somewhat better at preserving biological variation within different cell type/states compared to the other three methods (Fig. 2; Fig. S1, available as supplementary data at *Bioinformatics* online). Whereas cell types such as lymphocytes, macrophages, endothelial cells and interstitial cells are separated into well-defined clusters by all four methods, UMAP visualizations show that Scanorama, Seurat, and FastMNN have a tendency to overmix cells, thus resulting in poor representation of the transcriptional heterogeneity between healthy and diseased cell states.

**Table 1.** Benchmarking ComBat, Scanorama, Seurat, and FastMNN for scRNA-seq data integration using eight scIB metrics (Luecken *et al.* 2022).^a^

	Aortic valve dataset				Arabidopsis dataset			
	ComBat	Scanorama	Seurat	FastMNN	ComBat	Scanorama	Seurat	FastMNN
NMI_cluster/label	0.3694	0.3569	0.4807	0.3931	0.7384	0.7323	0.6981	0.5833
ARI_cluster/label	0.2013	0.2293	0.3753	0.3418	0.6299	0.6345	0.5655	0.4035
ASW_label	0.4974	0.5033	0.5327	0.5004	0.5568	0.5547	0.5542	0.5171
ASW_label/batch	0.8499	0.8892	0.9101	0.8907	0.9198	0.9044	0.8926	0.9083
Isolated F1	0.5191	0.4940	0.7298	0.4495	0.7496	0.8534	0.7470	0.7568
Isolated ASW	0.3942	0.4428	0.5640	0.4451	0.6278	0.6055	0.6297	0.5489
Graph Conn.	0.9404	0.9605	0.9882	0.9057	0.9486	0.9607	0.9613	0.9727
HVG Cons.	0.1340	0.0205	0.0340	0.0490	0.2697	0.0163	0.0654	0.0203
**Average score**	**0.4882**	**0.4871**	**0.5769**	**0.4969**	**0.6801**	**0.6577**	**0.6392**	**0.5889**

a A brief description of the metrics is given in Supplementary text, available as supplementary data at *Bioinformatics* online. Numbers in bold represent the average of the eight metrics for each method.

In this context, it should be noted that the Arabidopsis dataset consists of cells from only wild-type root samples (normal), whereas the aortic valve dataset consists of cells from healthy (normal) and diseased (abnormal) individuals. We therefore speculate that the sub-optimal performance of ComBat with the aortic valve dataset, as observed in Table 1, is perhaps due to the less aggressive cell mixing characteristics of the ComBat model, which in turn facilitates a better separation of healthy from diseased cells, even within the same cell type. Moreover, ComBat requires significantly less runtime and memory to integrate these scRNA-seq datasets when compared to Seurat and Scanorama (Table 2). We, therefore, decided to use the linear regression model as a basis for developing a faster and more efficient method for large-scale scRNA-seq integration.

**Table 2.** Runtime and memory usage of ComBat, Scanorama, Seurat, and FastMNN for scRNA-seq data integration.

	Runtime (s)		Memory (GB)	
	HAVD	AtRD	HAVD	AtRD
ComBat	172.81	66.71	19.41	6.11
Scanorama	530.75	125.63	38.34	13.24
Seurat	3298.44	1792.36	84.53	42.63
FastMNN	187.22	136.12	5.01	4.66

As our new parallel algorithm SCEMENT is built upon and extends the linear regression model previously applied in ComBat to an unsupervised sparse matrix setting, we first evaluated its integration performance in comparison to ComBat. As expected, our results show that there are no significant qualitative differences between ComBat and SCEMENT (Fig. S2, available as supplementary data at *Bioinformatics* online).

### 3.2 SCEMENT enables large-scale scRNA-seq integration

We assessed SCEMENT’s performance for large-scale integration of scRNA-seq datasets by measuring its runtime and memory usage with varying number of datasets/batches and cells. We used two sets of PBMC derived scRNA-seq datasets—205 samples from COVID-19 patients (Table 3) and another from 17 different healthy individuals (Table S2, available as supplementary data at *Bioinformatics* online). We performed batch correction and integration using intersection (genes common to all datasets) as well as the union of genes (all genes from all datasets) for all of the 8 subsets ranging from 3 to 205 COVID-19 datasets, and 7 subsets ranging from 2 to 17 healthy PBMC datasets. As expected, Table 3 and Table S2, available as supplementary data at *Bioinformatics* online, show that as the number of datasets increase, the number of intersecting genes decrease. In contrast, this number increases when we use the union of genes for constructing the integrated data.

**Table 3.** COVID-19 datasets from Ren *et al.* (2021).

No. of datasets	No. of cells	No. of genes intersection	No. of genes union
3	11 540	16 601	21 956
5	25 090	16 693	24 176
8	53 276	15 291	24 588
24	150 142	13 337	25 482
60	351 954	12 222	26 180
80	502 001	12 915	26 139
115	701 072	11 666	26 427
205	1 226 553	11 437	26 817

We assessed runtime and memory consumption of both the python (pySCEMENT) and the C++ (SCEMENT-CPP) versions of SCEMENT and compared these with three other methods—ComBat, Scanorama, and FastIntegration (Table S1, available as supplementary data at *Bioinformatics* online). ComBat uses the linear regression model for scRNA-seq data integration (Johnson *et al.* 2007), and Scanorama (Hie *et al.* 2019) and FastIntegration (Li *et al.* 2022a) have previously been shown to scale to million(s) of cells. FastIntegration is also a fast and high-capacity version of the Seurat integration tool. By default, currently available integration methods, including ComBat, Scanorama, and FastIntegration, generate an integrated data matrix containing cells with either a set of highly variable genes or the intersecting set of genes from all batches. However, in this study, we modified the ComBat workflow and applied the same preprocessing step as in the SCEMENT workflow (step P in Fig. 1) to generate an integrated data matrix containing union of genes using ComBat. Therefore, we include ComBat, but exclude FastIntegration and Scanorama from our comparisons involving union of genes.

**Figure 3.**
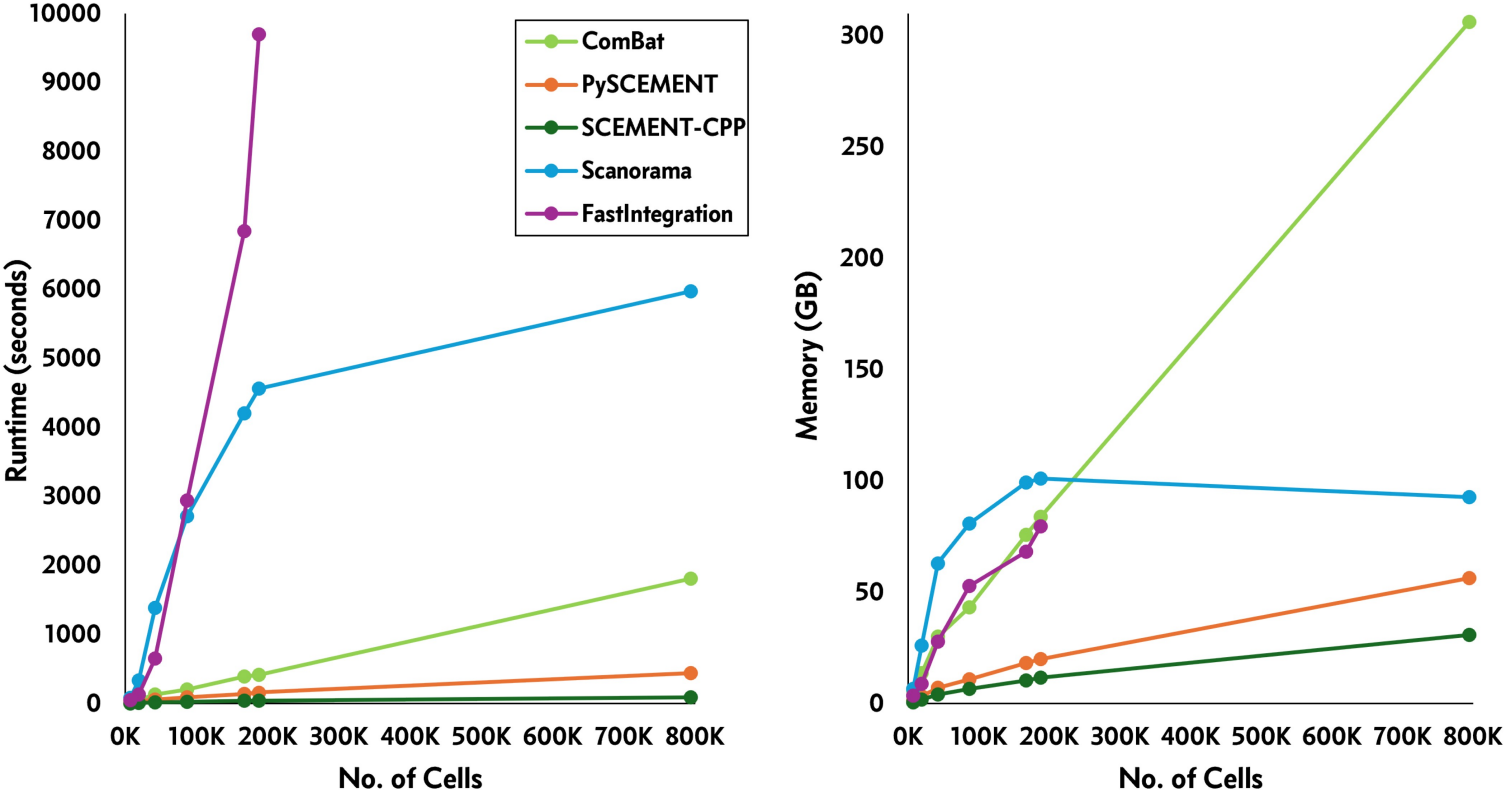
Runtime and memory usage of various scRNA-seq data integration methods for intersection of genes using 10× Genomics PBMC datasets.

**Figure 4.**
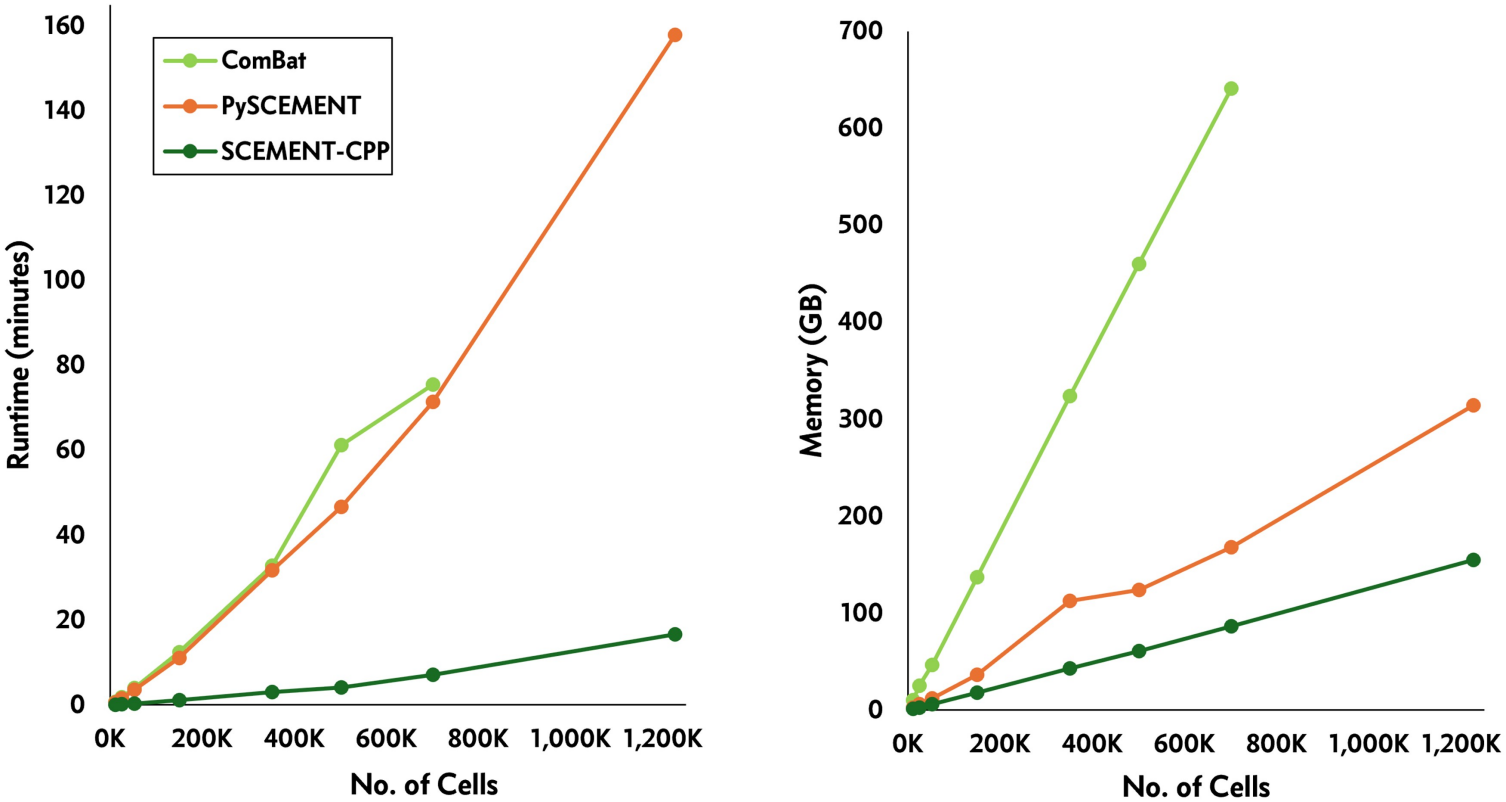
Runtime and memory usage of various scRNA-seq data integration methods for union of genes using COVID-19 datasets (Ren *et al.* 2021).

Our results show that SCEMENT-CPP outperforms all other methods in runtime and memory usage for both union as well as the intersection of genes (Figs 3 and 4; Figs S3 and S4, available as supplementary data at *Bioinformatics* online; Tables S4–S7, available as supplementary data at *Bioinformatics* online). It is upto 214× faster than FastIntegration, 106× faster than Scanorama, and 20× faster than ComBat depending on the number of datasets/cells/genes involved in the integration task. Moreover, SCEMENT-CPP uses upto 16× less memory than Scanorama and 10× less than ComBat, thus enabling integration of more than a million cells and more than 26 K genes in just 16–17 min. Even for smaller integration tasks for <200 K cells where all other methods are able to complete the integration task, SCEMENT is ∼10× times faster than ComBat, uses <20 GB of memory, and thus can accomplish this task on a modestly equipped workstation. Interestingly, ComBat and pySCEMENT perform comparably with respect to runtime, even though ComBat’s implementation is in parallel while pySCEMENT is sequential. pySCEMENT is also significantly more memory-efficient than ComBat. In fact, ComBat could not scale beyond 700 K cells for the union of genes as it runs out of memory available on our benchmarking hardware. This is because pySCEMENT uses a sparse matrix with 32-bit floating point option, while ComBat’s implementation uses the dense 64-bit matrix.

FastIntegration and Scanorama require significantly longer runtimes and more memory usage, respectively, when compared to SCEMENT and also ComBat (Figs 3 and 4; Tables S4–S7, available as supplementary data at *Bioinformatics* online). In our studies, Scanorama could not successfully complete the runs beyond ≈800 K cells for the intersection of genes. In addition, we (this study) and others have shown that FastIntegration can scale to millions of cells; however, it accomplishes large-scale integration by (i) splitting a large integration task into a number of smaller integration tasks, which it then successively integrates to build the final integrated matrix, (ii) restricting the data integration process to intersection of genes, and (iii) requiring each individual dataset to be small as it uses Seurat to process the datasets. Of the 17 different human PBMC datasets (Table S2, available as supplementary data at *Bioinformatics* online), one dataset contains a large number of cells (≈606 606 cells). In such a scenario, FastIntegration fails to complete the integration task.

To further assess SCEMENT’s scalability beyond a million cells, we applied it to a dataset of ≈4 million cells and 38 481 genes collected from 121 samples (Cao *et al.* 2020). Pre-processing and filtering of the data from 121 samples using *Scanpy* took ∼78 min for pySCEMENT and SCEMENT-CPP, and while both were able to successfully integrate data from all samples, SCEMENT-CPP was significantly faster than pySCEMENT and completed the run in just 22 min (Table S8, available as supplementary data at *Bioinformatics* online).

### 3.3 SCEMENT enables identification of rare cell types from large-scale scRNA-seq data

**Figure 5.**
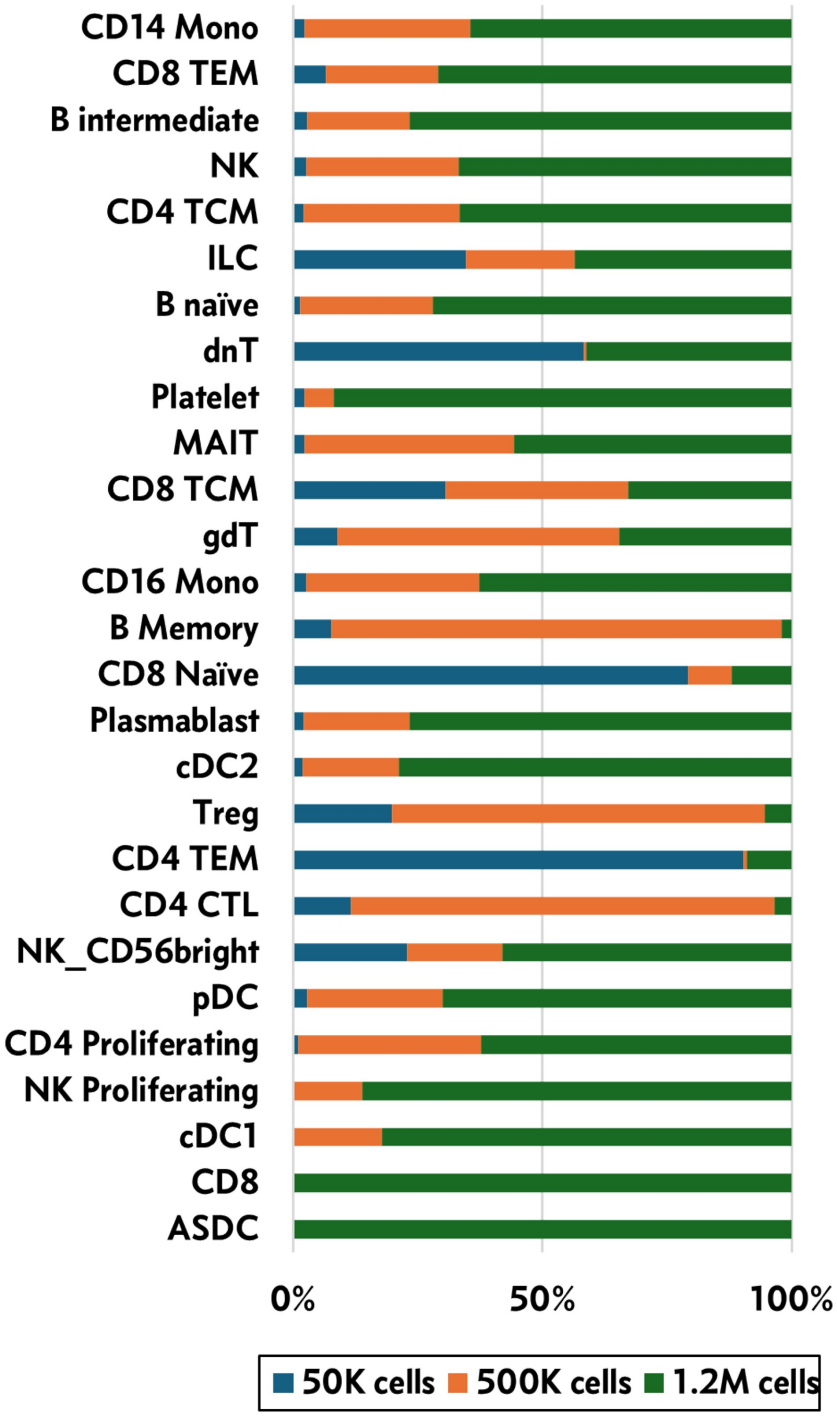
Stacked bar plot showing percentage of cells of each cell type in the 50 K, 500 K, and 1.2 million cell datasets. The CD8 and ASDC cell types are identifiable only in the 1.2 million dataset.

Large-scale scRNA-seq data analysis has been shown to facilitate a deeper understanding of the cellular heterogeneity and discovery of new rare cell types from complex tissues (Jindal *et al.* 2018, Qian *et al.* 2023). To assess whether SCEMENT enables improved identification of rare cell types, we used scRNA-seq data from PBMCs (Ren *et al.* 2021) and generated integrated matrices from three random subsets with cells ranging from ≈50 K to 1.2 million cells. The resulting matrices were subjected to automated cell-type identification using the Azimuth package. It is currently not feasible to run Azimuth on large data with full gene set. Therefore, we restricted the number of genes in the integrated matrices to only the top 1000 highly variable genes to make it feasible to perform cell-type annotation on large-scale data. Even with such limitations, our results show that the number of cell types identified increase with increasing number of cells (Fig. 5; Table S9, available as supplementary data at *Bioinformatics* online). Dendritic cells are the rarest cell types amongst the PBMCs (Patente *et al.* 2019). In our study, a minimum of 500 K cells were needed for discovery of conventional dendritic Cell 1 cells and more than a million for ASDC (AXL + dendritic cell) cells. It should be noted that such rare cell-type identification is feasible using only SCEMENT-CPP. As shown in Figs 3 and 4, FastIntegration and Scanorama either do not scale and/or require significantly longer runtimes and more memory usage for large data integration, while COMBAT runs out of memory for >700 K cells, especially for the union of genes.

### 3.4 SCEMENT facilitates robust GRN reconstruction from integrated scRNA-seq data

Reconstruction of GRNs from high-throughput gene expression (e.g., scRNA-seq) data requires quantitative gene expression information from a large number of genes and observations for determining accurate gene pair associations (Emmert-Streib *et al.* 2012). However, scRNA-seq data suffers from data sparsity, with each individual dataset containing gene expression profiles of a only a few thousand genes. Large-scale integration of multiple datasets may help overcome such limitations. It is currently not feasible to reconstruct GRNs from large-scale scRNA-seq data with hundreds of thousands to millions of cells and tens of thousands of genes, with existing GRN reconstruction methods. Therefore, to show utility of the integrated matrices and construct GRNs in a reasonable amount of time, we selected small-scale PBMC data with cells ranging from 20 to 166 K (Table S2, available as supplementary data at *Bioinformatics* online) and ≈2723 genes representing a non-redundant set of TFs and target genes from the TRRUSTv2 manually curated gene regulatory network (Table S3, available as supplementary data at *Bioinformatics* online). We then constructed GRNs from integrated data matrices containing the intersection (SCEMENT-CPP, ComBat, Scanorama, and FastIntegration) and the union set of genes (SCEMENT-CPP and ComBat) using the pySCENIC workflow (Kumar *et al.* 2021).

Network quality evaluation measures (Table 4, Table S10, available as supplementary data at *Bioinformatics* online) show that SCEMENT and ComBat are comparable in their performance with respect to recall, precision, and F-score values for the intersection of genes, with Scanorama and FastIntegration showing a 16% and 6% lower recall, respectively when compared to SCEMENT.

**Table 4.** Assessment of network quality.^a^

Method	Cells	Genes	Edges	Prec.	Recall	AUROC	AUPR
Intersection of genes							
SCEMENT	86 692	12 337	17 862	0.070	0.451	0.689	0.038
ComBat	86 685	12 337	17 460	0.068	0.445	0.689	0.039
Scanorama	79 528	12 337	17 131	0.070	0.374	0.657	0.034
FastIntegration	75 159	12 337	15 474	0.074	0.414	0.670	0.036
Union of genes							
SCEMENT	86 692	25 621	20 067	0.072	0.496	0.709	0.042
ComBat	86 692	25 621	19 787	0.074	0.500	0.711	0.043

a scRNA-seq data from nine different human PBMC datasets and cells totaling to ≈86 K was used to generate six different integrated matrices containing either the intersection or the union of genes. GRNs were then reconstructed from each of these integrated matrices using the pySCENIC workflow. Cells, Genes: Total No. of cells and genes used in network generation, respectively; Edges: total number of gene–gene interactions in the inferred network. Precision, Recall, AUROC, and AUPR: as defined in Section 2.3.1.

**Figure 6.**
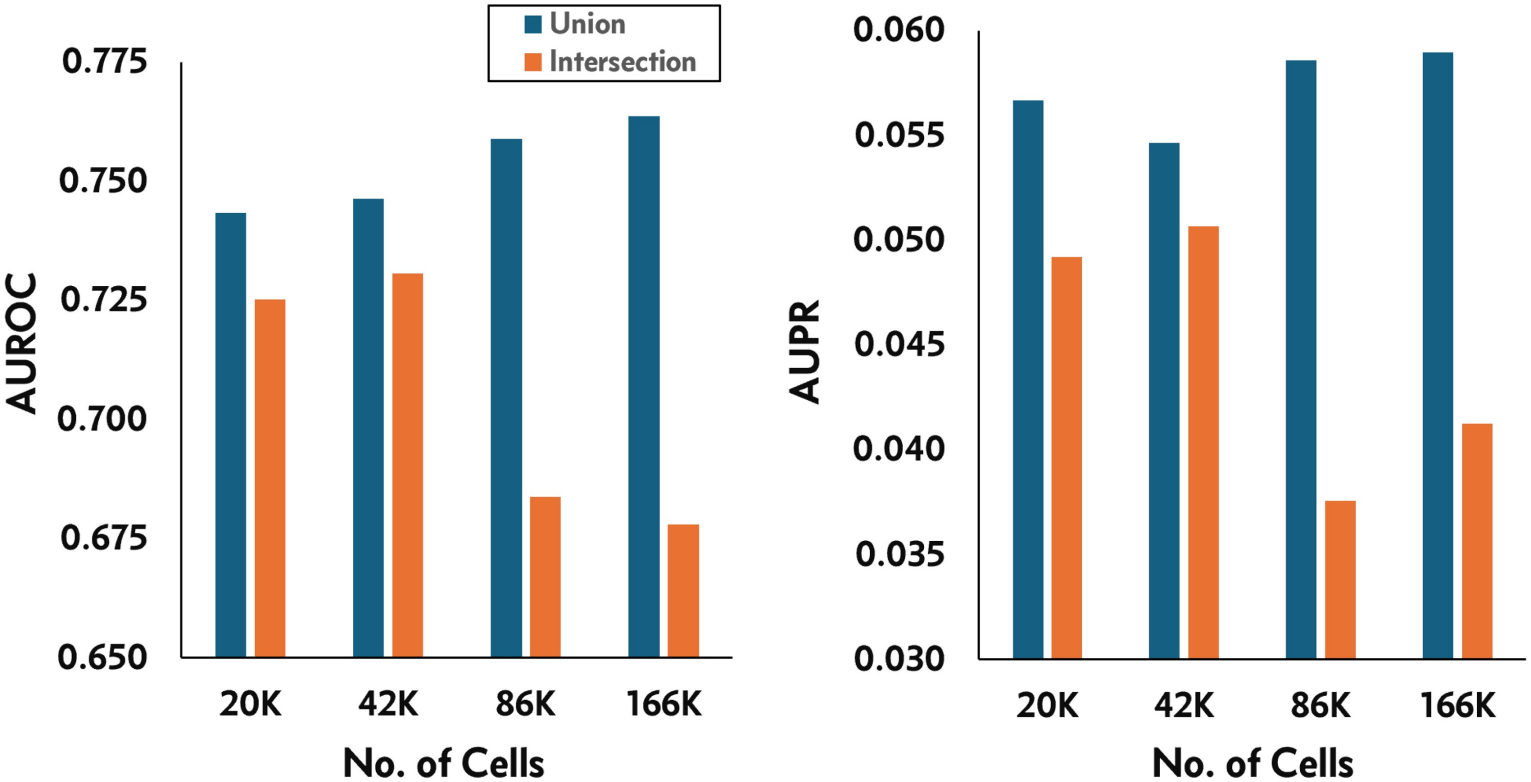
AUROC and AUPR for GRNs generated using the pySCENIC workflow from integrated PBMC 10× Genomics datasets with increasing number of cells. GRNs were constructed with integrated matrices containing either the union or intersection of genes.

The higher recall values in GRNs constructed from SCEMENT and ComBat also suggest less number of false positives in the network. More importantly, GRNs constructed from matrices containing the union set of genes show a significant improvement when compared to those containing intersection of genes—a 13% higher recall when compared to SCEMENT-intersection of genes, and more than 20%–30% improvement over FastIntegration and Scanorama. In addition, the union GRNs also show higher AUROC (4%–8%) and AUPR (11%–25%) values with SCEMENT suggesting that integrated matrices with union of genes result in more robust and accurate networks (Table S10, available as supplementary data at *Bioinformatics* online). In fact, our results show that the Recall, AUROC, and AUPR values increase with increasing number of cells and genes for networks containing the union of genes, while these measures decrease for larger networks reconstructed using the intersection of genes (Fig. 6 and Table S11, available as supplementary data at *Bioinformatics* online). In this context, it should be noted that as the number of datasets increase, the number of intersecting genes decrease (Table 3, Table S2, available as supplementary data at *Bioinformatics* online), which in turn reduces the number of genes available for GRN construction and hence, GRN accuracy. Overall, these results suggest that by incorporating gene expression profiles of all available genes from large number of input datasets in the integrated data matrix, SCEMENT enables more robust and accurate GRN reconstruction from single-cell data, even for small-scale GRNs.

## 4 Conclusions

Single-cell transcriptome analyses are hampered by data sparsity, and large-scale integration of scRNA-seq data can overcome these limitations to provide a more comprehensive understanding of the cellular heterogeneity. We have developed a fast, scalable, and memory-efficient method (SCEMENT) that enables accurate and large-scale integration of homogeneous and heterogeneous scRNA-seq datasets, and demonstrated its applicability on up to 4 million cells. SCEMENT is much faster and uses much less memory compared to existing methods. In fact, with SCEMENT, it is often not even necessary to have a high-memory system and an integration task of up to 500K cells and 25K genes can easily be completed on a laptop. We further demonstrate SCEMENT’s utility in the discovery of new and rare cell types, and for more accurate and robust reconstruction of large GRNs. Thus, SCEMENT is a simple but effective solution applicable to large- and genome-scale integration of multiple scRNA-seq datasets, and opens new avenues for data-driven construction of atlas-scale cell maps.

## Supplementary Material

btaf057_Supplementary_Data

## Data Availability

The data used for evaluation of the proposed method are available in Zenodo, at https://zenodo.org/doi/10.5281/zenodo.11521687, and the accession numbers and the data sources are listed in Zenodo.
