# Supplementary material for: SCEMENT: scalable and memory efficient integration of large-scale single-cell RNA-sequencing data
Source: Bioinformatics. 2025 Feb 22;41(2):btaf057. doi: 10.1093/bioinformatics/btaf057 (PMC12013815; doi:10.1093/bioinformatics/btaf057)
Supplement: btaf057_Supplementary_Data [file btaf057_supplementary_data.zip › Figure S1.pptx]

## Slide 1
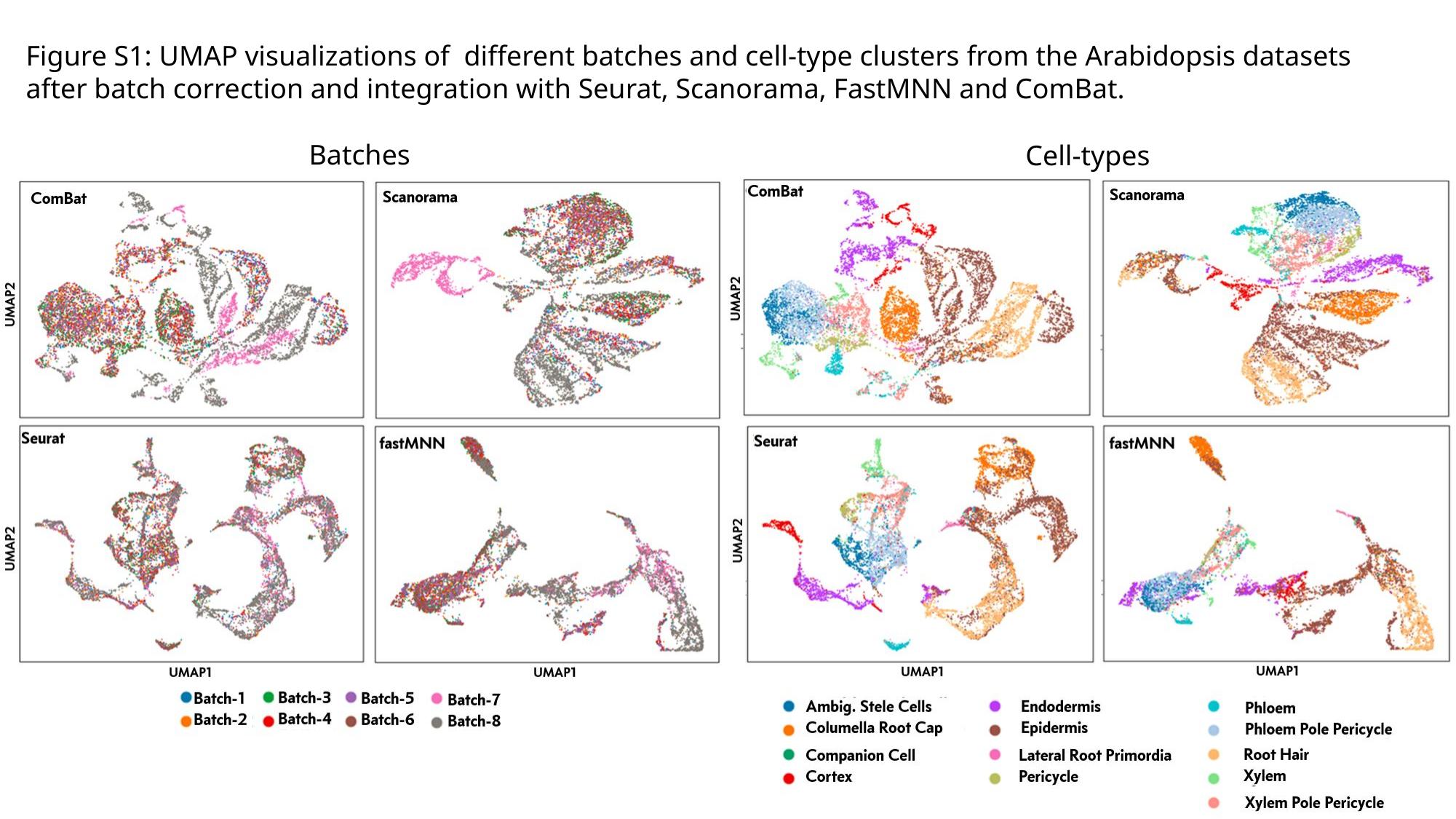

Figure S1: UMAP visualizations of  different batches and cell-type clusters from the Arabidopsis datasets
after batch correction and integration with Seurat, Scanorama, FastMNN and ComBat.
Batches
Cell-types
