# Supplementary material for: SCEMENT: scalable and memory efficient integration of large-scale single-cell RNA-sequencing data
Source: Bioinformatics. 2025 Feb 22;41(2):btaf057. doi: 10.1093/bioinformatics/btaf057 (PMC12013815; doi:10.1093/bioinformatics/btaf057)
Supplement: btaf057_Supplementary_Data [file btaf057_supplementary_data.zip › Figure S2.pptx]

## Slide 1
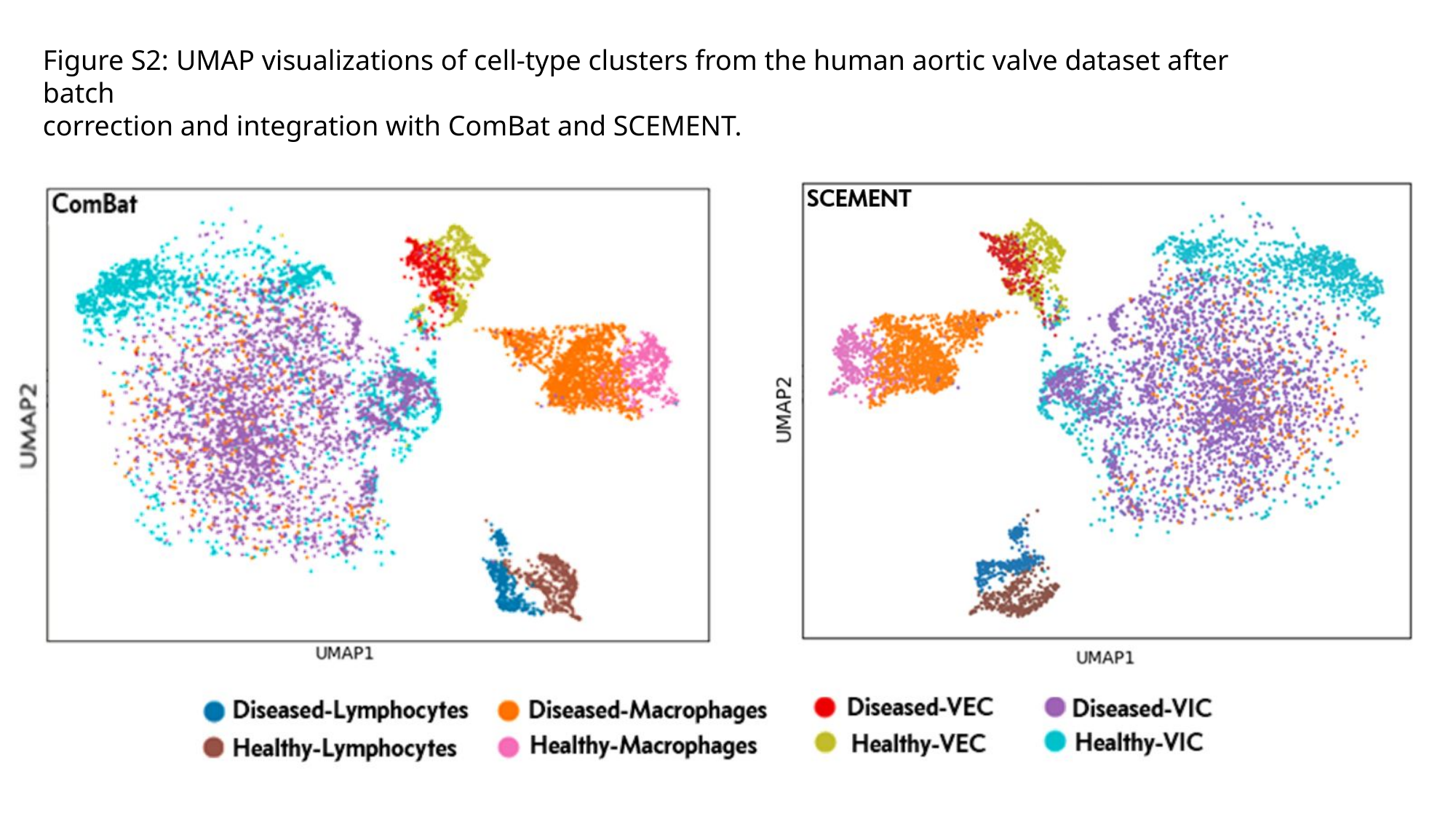

Figure S2: UMAP visualizations of cell-type clusters from the human aortic valve dataset after batch
correction and integration with ComBat and SCEMENT.
