# Supplementary material for: SCEMENT: scalable and memory efficient integration of large-scale single-cell RNA-sequencing data
Source: Bioinformatics. 2025 Feb 22;41(2):btaf057. doi: 10.1093/bioinformatics/btaf057 (PMC12013815; doi:10.1093/bioinformatics/btaf057)
Supplement: btaf057_Supplementary_Data [file btaf057_supplementary_data.zip › Figure S3.pptx]

## Slide 1
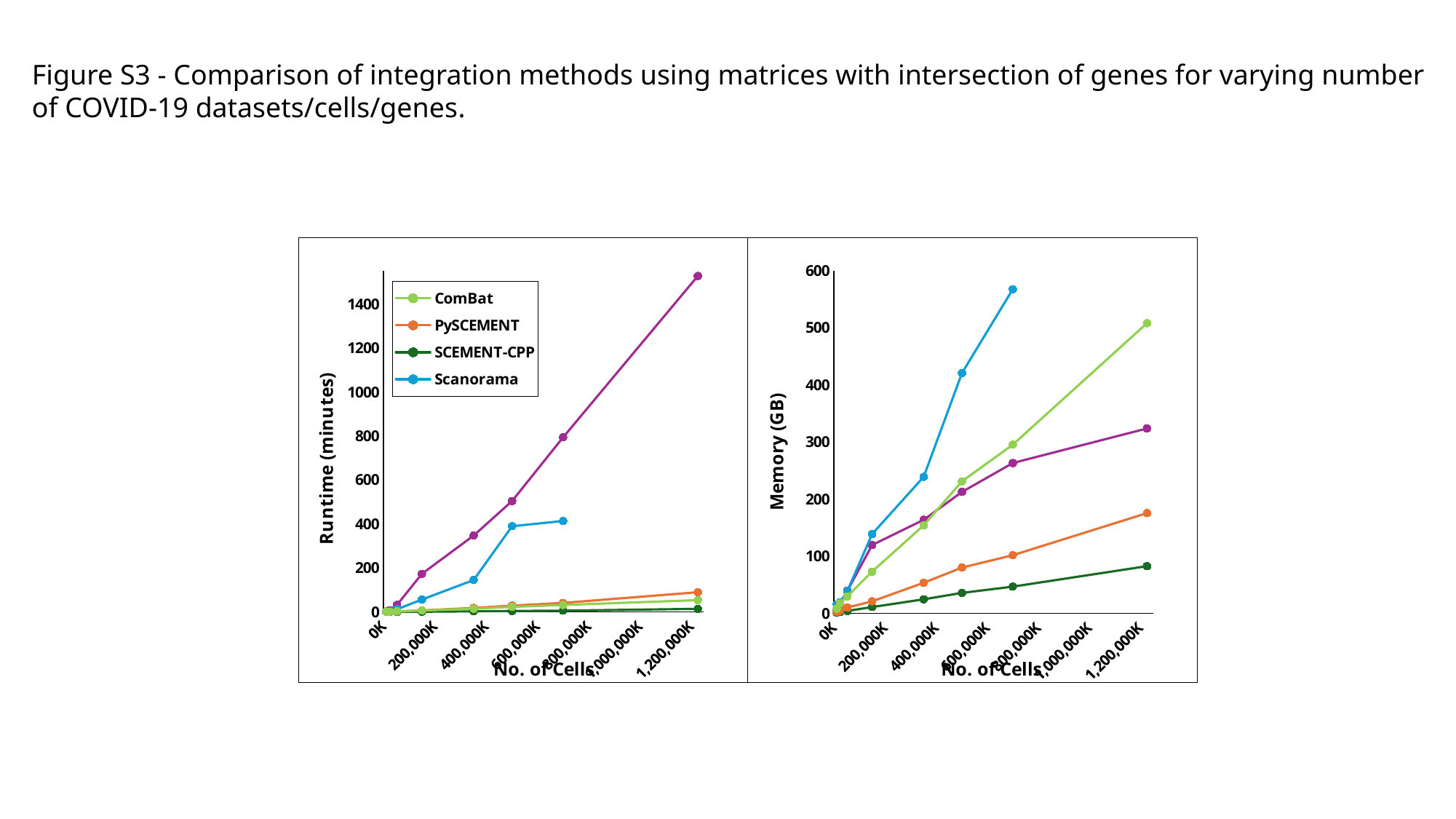

Figure S3 - Comparison of integration methods using matrices with intersection of genes for varying number
of COVID-19 datasets/cells/genes​.
### Chart
| Category | ComBat | PySCEMENT | SCEMENT-CPP | Scanorama | FastIntegration |
|---|---|---|---|---|---|
### Chart
| Category | ComBat | PySCEMENT | SCEMENT-CPP | Scanorama | FastIntegration |
|---|---|---|---|---|---|
