# Supplementary material for: SCEMENT: scalable and memory efficient integration of large-scale single-cell RNA-sequencing data
Source: Bioinformatics. 2025 Feb 22;41(2):btaf057. doi: 10.1093/bioinformatics/btaf057 (PMC12013815; doi:10.1093/bioinformatics/btaf057)
Supplement: btaf057_Supplementary_Data [file btaf057_supplementary_data.zip › Figure S4.pptx]

## Slide 1
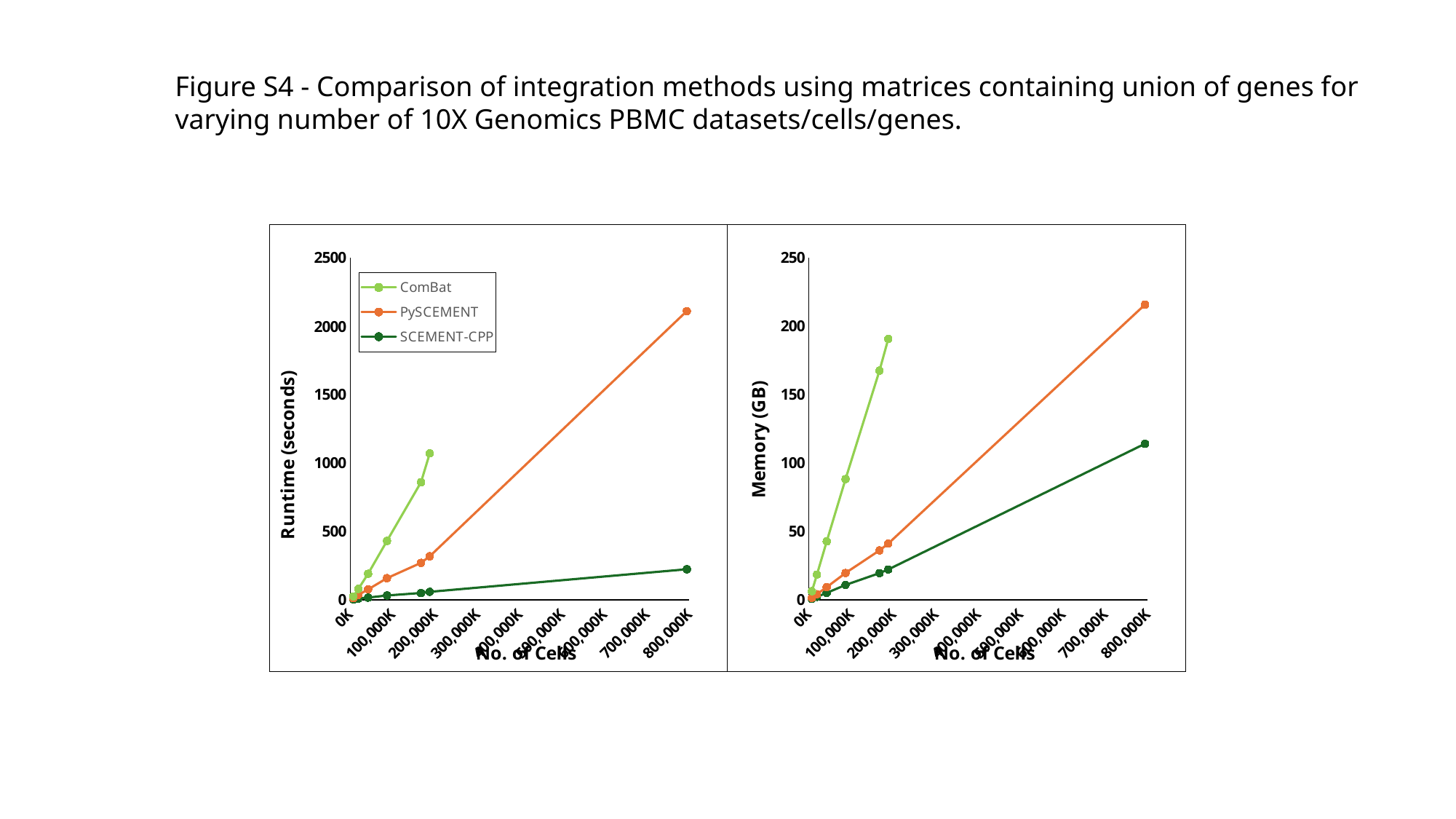

Figure S4 - Comparison of integration methods using matrices containing union of genes for
varying number of 10X Genomics PBMC datasets/cells/genes.
### Chart
| Category | ComBat | PySCEMENT | SCEMENT-CPP |
|---|---|---|---|
### Chart
| Category | ComBat | PySCEMENT | SCEMENT-CPP |
|---|---|---|---|
