# Supplementary material for: SCEMENT: scalable and memory efficient integration of large-scale single-cell RNA-sequencing data
Source: Bioinformatics. 2025 Feb 22;41(2):btaf057. doi: 10.1093/bioinformatics/btaf057 (PMC12013815; doi:10.1093/bioinformatics/btaf057)
Supplement: btaf057_Supplementary_Data [file btaf057_supplementary_data.zip › SCEMENT_S1.pdf]

S1 Supplementary Text

S1.1 Empirical Bayes Model

The linear model for integration used in ComBat is represented by

$$X = \alpha + D\beta + \gamma + \delta\epsilon$$

where gene expression data is modeled as a function of the four terms.

- The average or overall gene expression, denoted by α, is an $m \times n$ matrix. Each row i in α corresponds to the gene expression profile of the set of conditions the cell i belongs to.
- Linear combination of the independent variables, β. It is the matrix of regression coefficients (size $c \times n$) with each column corresponding to a specific variable. In case of integration, the independent variables are the condition/batch variables. D is the design matrix, a binary $m \times c$ matrix such that entry $D[i, j]$ is 1 if input i is observed under the condition j.
- Additive batch effect denoted by γ.
- Multiplicative batch effect denoted by δ. Furthermore, ϵ is the error term, assumed to follow a normal distribution with variance σ, i.e, $\epsilon \sim \mathcal{N}(0, \sigma^2 I_n)$.

The Empirical Bayes based batch correction, as developed in COMBAT Johnson *et al.* (2007), for the above linear model proceeds with the following three steps:

1. Compute estimates $\hat{\beta}$, $\hat{\alpha}$ and standardize the input matrix.
2. Iteratively update to estimate batch effect δ.
3. Compute the batch corrected matrix X'.

S1.1.1 Standardizing data

A standardized matrix is constructed first and used in all the subsequent steps since it provides well-behaved mean and variance characteristics for more robust downstream analyses.

A single sample-type can be observed simultaneously under multiple conditions. For example, a plant leaf sample belonging to a specific genotype can be observed under low and high stress conditions. Row i in the D matrix corresponds to the set of conditions under which sample i is observed. We call this row as the condition-profile of sample i. In the case when a sample in X belongs to exactly one condition, the least squares estimates for $\hat{\beta}$ and $\hat{\alpha}$ are

$$\hat{\beta} = (D^T D)^{-1} D^T X \quad \text{and} \quad \hat{\alpha} = \frac{1}{n} \mathbb{1}_m \cdot (N_c^T \hat{\beta})$$

respectively, where $N_c = [n_1, \ldots, n_c]^T$ is a vector with the number of observations in each condition, and $\mathbb{1}_m$ is m-length vector of ones. The estimate for the variance vector $\hat{\sigma}^2$ is

$$\hat{\sigma}^2 = \frac{1}{m}(X - D^T \hat{\beta})^{\odot 2} \cdot \mathbb{1}_m,$$

where the notation $A^{\odot 2}$ represents the Hadamard product of the matrix A with itself, i.e., the entry $A^{\odot 2}[i, j]$ is $(A[i, j])^2$ and the dot product $A \cdot 1_m$ indicates the sum across the columns of A.

After estimating $\hat{\alpha}$ and $\hat{\sigma}$, the standardized matrix Z is constructed as

$$Z = \frac{X - \hat{\alpha}^T}{\hat{\sigma} \cdot \mathbb{1}_n}$$

In the above equation, we use the division operation of two matrices of same sizes to indicate an element-wise division of numerator matrix entry to the denominator matrix entry.

In cases when a sample is subjected to multiple different conditions, let S_c be the set of conditions that divides the dataset into b partitions, i.e., $|S_c| = b$. In single-cell experiments, this can be the batch a sample belongs to. We assume that the first b columns of D correspond to these b conditions. In this case, the estimates of $\hat{\beta}$ and $\hat{\sigma}^2$ remain the same as the first case, while $\hat{\alpha}$ is computed as follows:

$$\hat{\alpha} = \left(\frac{1}{n} \mathbb{1}_m \cdot (N_b^T \hat{\beta}_b) \right) + D_{-b} \hat{\beta},$$

where $\hat{\beta}_b$ is a $b \times m$ sub-matrix of $\hat{\beta}$ such that $\hat{\beta}_b = \hat{\beta}[(1, \ldots, b);\ (1, \ldots, n)]$ and D_{-b} is D with the entries corresponding to the batch rows set to 0, i.e., $D_{-b}[(1, \ldots, m); (1, \ldots, b)] = 0$.

S1.1.2 Iterative Algorithm for Batch update

Given that the dataset X is merged from b batches, then Z, in this case, is a standardized matrix that takes into account the batch weights. The linear model assumes that, for a given batch i and gene g, the additive and multiplicative effects γ_{ig} and δ_{ig} are drawn from $\mathcal{N}(X_i, t_i^2)$ and Inverse Gamma(a_i, b_i) distributions respectively.

An empirical Bayes approach to estimate these parameters lends itself to an iterative solution. In iteration $k+1$, the estimates for each batch i are updated based on the estimates of the previous iteration k ($\hat{\gamma_i}^{(k)}$ and $\hat{\delta_i}^{(k)}$) as follows Johnson *et al.* (2007). First, $\hat{\gamma_i}^{(k+1)}$ is computed as $\hat{\gamma_i}^{(k+1)} \leftarrow (t_i n \hat{\gamma_i}^{(k)})/(t_i n + \hat{\delta_i}^{(k)})$. Then, $\hat{\delta}_i$ is updated as

$$\hat{\delta}_i^{(k+1)} \leftarrow \frac{(2 + a - 1)}{2n} \left(b + \left(Z - \hat{\gamma}_i^{(k+1)^T} \cdot \mathbb{1} \right)^{\odot 2} \cdot \mathbb{1} \right),$$

where the dot product with $\mathbb{1}$ indicates the row-wise summing operation. The above steps are repeated until the percentage change of both $\hat{\gamma}$ and $\hat{\delta}$ is less than the required tolerance.

S1.1.3 Batch Corrected Matrix

After the α, β, δ, and σ values are computed, the final step is to update the input gene expression matrix X_b.

$$X_b \leftarrow \hat{\alpha} + X\hat{\beta} + \frac{\hat{\sigma}}{\hat{\delta}}(Z_i - \hat{\gamma}_i)$$

S1.2 Metrics for Batch Integration

1. *NMI* and *ARI* compare the overlap of clustering with respect to the cell-type labels, with 0 being bad overlap and 1 being perfect match.
2. *ASW* measures the separation of clusters where 1 denotes dense and well-separated clusters, while 0 or -1 represents overlapping clusters.
3. *Isolated* scores were developed by Luecken *et al.* (2022) to evaluate how well data integration methods handle cell-types that appear in few batches.
4. *Graph Conn.* metric assesses how well the kNN graph constructed from integrated data directly connects all the cells of the same cell-type.
5. *HVG Cons.* score is a proxy for the preservation of highly variable genes after integration.
